# Assessment of maternal health services: a comparative study of urban and rural primary health facilities in Kano State, Northwest Nigeria

**DOI:** 10.11604/pamj.2021.38.320.25214

**Published:** 2021-03-30

**Authors:** Badia Maje Sayyadi, Awwal Umar Gajida, Rahila Garba, Usman Muhammad Ibrahim

**Affiliations:** 1 Department of Obstetrics and Gynecology, Muhammad Abdullahi Wase Teaching Hospital, Kano, Nigeria,; 2 Department of Community Medicine, Bayero University, Kano/Aminu Kano Teaching Hospital, Kano, Nigeria,; 3 Department of Community Medicine, Aminu Kano Teaching Hospital, Kano, Nigeria

**Keywords:** Maternal, services, rural, urban, quality

## Abstract

**Introduction:**

all pregnant women are at potential risk of obstetric complications; majority of which can be treated if appropriate care is accessed promptly. A shift in focus to quality of care has the potential to unlock significant returns for every mother and newborn to end preventable maternal and infant deaths. The study aimed to assess the quality of maternal health services in primary health facilities in urban and rural communities of Kano State.

**Methods:**

using a comparative cross-sectional study design that utilized mixed method of data collection, interviewer administered questionnaire were used to collect information from 438 women (219) each attending health facilities for maternal health services in rural and urban areas of Kano using multistage sampling technique from June to November, 2019. Six Key Informant Interviews with the heads of units/ facilities were purposively conducted. Quality of care was assessed using perspectives and system models based on the components of antenatal care received, postnatal care and perception of care received. A statistical significance was set at p-value < 0.05. Thematic framework analysis was used to analyze verbatim transcript from qualitative interviews.

**Results:**

the age of the respondents ranged from 18-48 years with majority having secondary education in both communities. In both urban and rural communities, majority of the respondents had only 1-3 antenatal care visits making up 63.5% and 70.3% respectively. Almost similar proportions of the urban (58.4%) and rural (50.2%) respondents were delivered by a skilled birth attendant. About two-third of the respondents, 67.6% and 65.3% in the urban and rural communities respectively were completely satisfied with the quality of care received. Qualitative interviews pointed ignorance as the major factor that prevent mothers from accessing quality care and reported that satisfactory services were provided in all facilities.

**Conclusion:**

considerable disparity exists between urban and rural communities in quality of maternal health services with better provision of most services in the urban communities. There is need for improvement in the desirable and minimum acceptable quality of maternal health services in Kano State.

## Introduction

Maternal health care services constitute a range of curative and preventive health services of particular importance to the health of women of reproductive age and includes population-based services such as behavior change and health communication [1]. Maternal health services aim at reducing maternal mortality and morbidity by ensuring pregnant women remain healthy throughout pregnancy, deliver safely to healthy babies and recover fully from the physiological changes that occur during pregnancy [2]. The quality of maternal care is a profound factor that impacts on the delivery of continuum of care among women in most of the sub-Saharan African countries and this is attributed to existence of inequalities within and between developing countries the way operational management of health care services is implemented [3], this situation subsequently affects quality of care and it increases the women´s use of alternative health care services. In order to reduce the number of women that do not use health facilities during pregnancy and delivery there is need to improve coverage of health facilities that provide skilled delivery care especially in rural areas [3].

The WHO Quality of Care Framework outlines various components of quality of care for maternal health, a few of which fall under the heading of patient experiences [4]. Research has shown that women´s decisions about where they will go for care is influenced by the types of interactions they have with providers in the past and their perceptions of quality of care provided [4]. Quality care can be viewed as both a desired goal and so used to judge the health system, or as an intervention for achieving health outcomes, such as improved survival, in both cases, the major evidence gap is how to implement care that is clinically effective, safe and a good experience for the patient [5]. The Sustainable Development Goals have set ambitious health -related targets for mothers, newborn and children under the umbrella of Universal Health Coverage by 2030 and addressing the quality of care will be fundamental in reducing maternal mortality and achieving the health-related SDG targets. In this context, the World Health Organization (WHO) has elaborated a global vision where “every pregnant woman receives quality care throughout pregnancy, childbirth and the postnatal period [6]”.

In Nigeria less than 5% of antenatal care (ANC) users receive desirable quality of services with about one tenth receiving minimum acceptable quality [7]. In addition, place of residence and zones significantly the receipt of good quality antenatal care [7]. Rural-Urban differences and geographical location of residence also influenced receiving good quality with mothers in rural areas having lower odds of receiving desirable quality of care compared to those in the urban areas. In Nigeria, study reported adequate utilization of maternal health services, thus the problem of unacceptable high maternal mortality in Nigeria was attributed not necessarily to utilization but to quality of services [8]. A wealth of knowledge and experience in enhancing the quality of healthcare has accumulated over many decades. In spite of this wealth of experience, the problem frequently faced by policymakers at country level in both high- and low-middle income countries is to know which quality strategies complemented by and integrated with existent strategic initiatives would have the greatest impact on the outcomes delivered by their health systems [9]. This study assessed the quality of maternal health services in urban and rural communities of Kano State as it is important locally to assess the quality of care of maternal services to generate hypotheses to guide interventions and make recommendations that will reduce maternal morbidity and mortality and therefore improve general well-being of the population and identify areas that need improvement or alternative measures. Additionally, it is necessary to periodically determine the quality of care provided in our facilities at all levels until we reach and maintain the desired acceptable level of care for maternal health services.

## Methods

**Study area/setting:** Kano State is located in Northern Nigeria and lies between 12000 North and longitude 8031´ East. According to 2006 census, the population of Kano State was 9.6 million [10]. At a projected increase of 3% per annum the estimated population is 13,377,462 in the year 2017 [11]. The total land area of Kano State is 20,760sqkm and is the most densely populated state in Northern Nigeria with over 300 persons per square kilometer. The population is predominantly rural although around one-quarter of the populace lives in urban areas, mainly in the metropolis. Six out of the 44 Local Government Areas were selected; three each from the urban and rural LGAs. The urban local governments are; Tarauni, Gwale and Nassarawa while the rural local governments are; Gezawa, Minjibir and Dawakin Kudu. Hausa and Fulani inhabit the state predominantly and although Hausa is the predominant language spoken by indigenes and non-indigenes, English is the official language. The main religion practiced is Islam but there is a significant Christian population mostly from other states. Maternal Health Services are provided at the three levels of care; primary, secondary and tertiary. The State Ministry of Health and its parastatals; the Hospitals Management Board and Primary Healthcare Management Board manage the facilities at the state and local government levels while the three federal tertiary health facilities namely Aminu Kano Teaching Hospital, National Orthopaedic Hospital Dala and Armed Forces Specialist Hospital provide maternal health services at the Federal level. There are also several private clinics, pharmacy stores and patent medicine stores especially in the metropolitan local government areas.

**Study design:** comparative cross-sectional study design with concurrent mixed method of data collection (Quantitative and Qualitative) was used.

**Study population:** the study population comprised of women accessing maternal health care at the antenatal/postnatal clinic for the quantitative arm, while healthcare workers in charge of the facilities/units were selected for the qualitative arm of the study. Women residing for at least 1 year within the study areas who have had at least three contacts with the health facility within the preceding one year were included in the study while non-consenting women and women that were sick were excluded.

**Sample size determination:** a sample of 219 participants each from urban and rural LGA was determined using the formula for comparison of proportion [12].

$$\text{n=}\frac{{\left({\text{Z}}_{\alpha }+Z_{1-\beta }\right)}^{2}\left[{\text{P}}_{1}(1-{\text{P}}_{\text{1}})+{\text{P}}_{\text{2}}(1-{\text{P}}_{\text{2}})\right]}{{\left({\text{P}}_{1}-{\text{P}}_{\text{2}}\right)}^{2}}$$

Substituting the values, Z_α_=Value of standard normal deviate corresponding to 95% confidence interval=1.96, Z_1-ß_= Standard normal deviate corresponding to a power of 80% (obtained from normal distribution table)=0.84, P_1_=Proportion of ANC users in urban communities receiving minimum acceptable quality of care obtained from a previous study in Nigeria [13] = 50.8% P_2_ = Proportion of ANC users in rural communities receiving minimum acceptable quality of care from a previous study in Nigeria [13]= 37.0% and possible non-response rate of 10% were used to calculate the sample size. For the qualitative arm, providers of various cadres including midwives, nurses and community health workers involved in the provision of maternal health services were interviewed from the selected facilities to collect information. Six Key Informant Interviews involving the hospital in charge/maternal services provider were conducted, one in the selected rural and urban facilities of the selected wards.

**Sampling technique:** a multi-stage sampling technique was employed to select the respondents. Stage 1- Three urban and three rural local governments´ areas (LGA) out of the 44 LGAs in Kano State were randomly selected using balloting. Stage 2- One political ward from each of the 6 LGAs with health facilities was selected randomly using simple random sampling technique specifically balloting. Stage 3- One facility providing full range of maternal health service was selected from each of the selected wards using balloting. Stage 4- Respondents were selected proportionately from each health facility. Systematic sampling technique was used at this stage. Using the allocated sample for the facility and average antenatal care attendance for the month preceding the survey, as sampling frame, sampling interval was calculated as the ratio of sampling frame to the sample size. The first respondent in each of the selected facility was randomly selected by balloting using the numbers within the sampling interval, thereafter; subsequent respondents in each of the selected facilities were selected by adding the sampling interval of the facility until the proportionately allocated sample size was obtained. Key informants were either the facility in charges or in charge of maternal services and were purposively selected for the interviews.

**Method of data collection:** data was collected using an adapted structured interviewer administered questionnaire [14] and Key Informant Interview Guide. The questionnaire was pretested on a similar populace from different urban and rural communities (Kano Municipal and Kura L.G.As). Six community health extension workers were the research assistants and were trained for three days to assist in administering questionnaires. They were trained on the research objectives, how to administer the tools and the ethical issues. Identification numbers were used on each questionnaire and linked to each respondent´s anonymous data entry. Qualitative data was collected using Key Informant Interviews (KII) of delivery service providers. The interviews were held with delivery service providers who were selected using purposive sampling. A note taker assisted with taking notes on verbal and non-verbal responses and recorded the discussion.

**Data management and analysis:** data was analyzed using SPSS version 21 for Windows. Quantitative variables were summarized using mean and standard deviation or median and range as appropriate while qualitative variables were summarized using percentages and proportions. Quality of care was assessed using perspectives and system models based on the components of antenatal care received, postnatal care and perception of care received, [15,16] at bi-variate level, chi-squared test was used to assess relationship between quality of services provided at 5% α- level of significance. Key Informant interviews were analyzed manually using thematic framework analysis.

**Ethical considerations:** ethical approval was obtained from Aminu Kano Teaching Hospital Research Ethics Committee with approval number NHREC/21/08/2008/AKTH/EC/2518 dated 31^st^ May, 2019 and MOG/OFF/797/230 dated 7^th^ May, 2019 respectively. Permission to conduct the study was obtained from the Kano State Ministry of Health, Kano State Primary Health Care Management Board and the selected local governments. All necessary information was explained to the potential participants assuring them of their confidentiality, anonymity and freedom to withdraw at any time without any negative consequence and their informed consent was obtained. Once understood the participants signed or thumb printed the consent form depending on their literacy level. A witness to the informed consent also countersigned the form. Data was collected between 1^st^ June, 2019 to 2^nd^ November, 2019. All the principles of research ethics were adhered to throughout the study period.

## Results

**Socio-demographic characteristics of respondents:** the age of the respondents ranged from 18 years to 48years with a mean of 30.2±5.56 years and 28.9±6.46 years among the urban and rural respondents respectively. Similarly, majority had a parity of 1-4, 54.3% and 48.9% in both urban and rural communities; with a mean parity of 3.62 ±1.58 and 3.52 ±1.74 respectively. Majority of the respondents, 48.4% and 41.1% had secondary education in both urban and rural communities. In both the urban and rural communities, majority of the respondents had delivered 24 months or less accounting for 53.4% and 66.6% respectively as shown in Table 1.

**Table 1:** socio-demographic characteristics of the respondents

Characteristics	Urban (n =219) n (%)	Rural (n =219) n (%)
**Age group**		
<20	9(4.1)	23(10.5)
20-29	102 (46.6)	111(50.7)
30-39	94 (42.9)	64(29.2)
≥40	14 (6.4)	21(9.6)
**Parity**		
0	16 (7.3)	22(10.0)
1-4	119 (54.3)	107(48.9)
>4	84 (38.4)	9**0**(41.1)
**Occupation**		
Trading	98 (44.7)	70(31.9)
Civil servant	10 (4.6)	5 (2.3)
Unemployed	80 (36.5)	99(45.2)
Others	31 (14.2)	45(20.6)
**Educational status**		
No formal education	26(11.9)	41(18.7)
Primary	39(17.8)	65(29.7)
Secondary	106(48.4)	90(41.1)
Tertiary	48(21.9)	23(10.5)
**Duration since last child birth**		
≤24 months	117 (53.4)	146 (66.7)
>24 months	102 (46.6)	73 (33.3)
**Husbands***´* **occupation**		
Trading	98(44.7)	94 (42.9)
Farming	10 (4.6)	56 (25.6)
Civil servant	53(24.2)	25(11.4)
Unemployed	5(2.3)	6(2.7)
Others	53(24.2)	38(17.4)

**Quality of maternal health services among respondents:** in both the urban and rural communities, majority of the respondents had only 1-3 visits for antenatal care making up 63.5% and 70.3% respectively. More than a half of the respondents, 59.4% and 64.8% had booked for antenatal care after 20 weeks of gestation in the urban and rural communities respectively with no statistically significant association (p > 0.05) as shown in Table 2. Only 62.6% of the urban respondents had blood pressure measurement during antenatal care compared to 92.7% in the rural communities and the difference is statistically significant (p < 0.05) Similarly, more than 90% of the respondents in both urban and rural communities had routine investigations done and received medications during antenatal care. About one-half 49.3% and 57.5% of the respondents had received information about warning signs in the urban and rural communities respectively. Majority of the respondents, 80.8% in the urban communities had received postnatal care compared to 76.3% in the rural communities with no statistically significant difference (p > 0.05). Similarly, over 70% of the respondents had been examined during their visits in both communities with no statistically significant difference (p > 0.05). Only 6.8% in the urban communities had used contraception compared to 4.1% in the rural communities (p > 0.05). Likewise, only 2.7% and 0.9% of the respondents had Pap smear taken in the urban and rural communities (p < 0.05).

**Table 2:** quality of maternal health services

	Urban n (%) n=219	Rural n (%) n=219	χ2	p-value
**Frequency of antenatal visits**				
1-3	139 (63.5)	154 (70.3)	2.64	0.104
≥4	80 (36.5)	65 (29.7)		
**Timing of booking**				
<20weeks	89 (40.6)	77 (35.1)	1.397	0.237
≥ 20weeks	130 (59.3)	142 (64.8)		
**Components of antenatal care received**				
Blood pressure taken	137 (62.6)	203 (92.7)	52.261	**0.000**
Routine Investigations	200 (91.3)	207 (94.5)	1.701	0.192
Routine drugs	208 (94.9)	211 (96.3)	0.495	0.482
Information on warning signs	108 (49.3)	126 (57.5)	2.973	0.085
Counseling	156 (71.2)	108(49.3)	22.84	**0.000**
Tetanus Toxoid Immunization	182 (83.1)	122(55.7)	38.70	**0.000**
**Delivery by skilled birth attendant**				
Yes	128 (58.4)	110(50.2)	2.837	0.092
No	91 (41.6)	109(49.8)		
**Received postnatal care**				
Yes	177(80.8)	167 (76.3)	1.355	0.244
No	42(19.2)	52 (23.7)	0.217	0.642
Physical examination	170(77.6)	174(79.5)	1.282	0.735
Contraception	15(6.8)	9(4.1)	1.616	0.204
Pap smear	6(2.7)	2(0.9)	409.6	**0.000**

**Figure 1:**
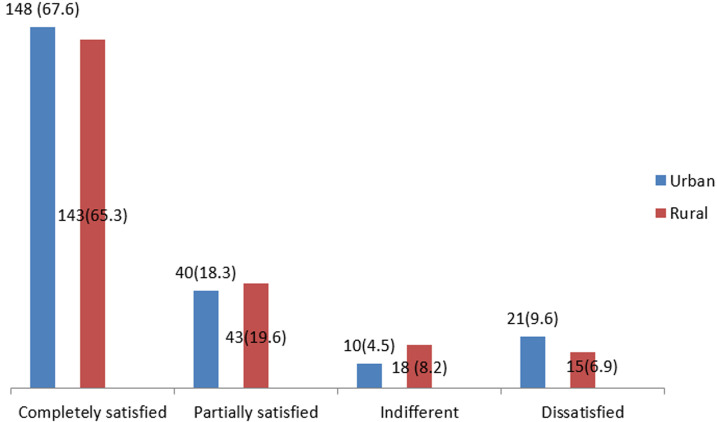
satisfaction of clients with overall quality of maternal health services (χ^2^= 0.07, p = 0.8)

**Satisfaction of clients with quality of delivery services:** majority of the women (70.3% and 63.0%) were completely satisfied with the delivery services in the urban and rural communities respectively compared to 9.1% and 14.6% who were dissatisfied with the delivery services as shown in Table 3. About two-thirds of the clients, 67.6% and 65.3%, in both the urban and rural communities respectively were completely satisfied with the quality of care received. On the other hand, about 9% were dissatisfied with the care received compared to 6.9% in the rural communities as shown in Figure 1.

**Table 3:** satisfaction of clients with quality of delivery services

	Urban n (%) n=219	Rural n (%)n=219	χ2 test	p-value
**Satisfaction with delivery services**				
Completely satisfied	154 (70.3)	138 (65.3)		
Partially satisfied	39 (17.8)	37 (16.9)		
Indifferent	6 (2.8)	12 (5.5)	0.327	0.960
Dissatisfied	20 (9.1)	32 (14.6)		

**Key informant interviews:** the key informants’ narratives were based on the below thematic areas.

**Quality of maternal health services:** all the health workers in both the urban and rural communities were of the opinion that they provide satisfactory quality of maternal health services in their facilities. They indicated during the interviews that maternal health services were provided in all the facilities ranging from antenatal care services, skilled delivery, and postnatal care to contraceptive services. One respondent said: *´Services provided are similar in all facilities. The Government, Non-governmental organizations and philanthropists provide equipment but we need more manpower, equipment and supplies to cater for the number of clients requiring services.´* (37-year-old midwife). Another respondent described quality as *´the provision of adequate and satisfactory services and timely referral of complicated cases that cannot be handled immediately´* (42-year-old CHEW). In line with this, another respondent said, *´there is need for health education and awareness to encourage women to access the care available´* (42-year-old nurse). The difference basically between most facilities is the provision of 24-hours services thus some patients with complications need to be referred.

**Perception of quality of care:** the first question under this theme was to ascertain what is believed to be the perception of quality of maternal health services; what quality of care means for the communities and patients that depend on it, the health care providers who provide it and the managers and administrators who oversee it based on the perspective model of care. All of the respondents described their provision of care as satisfactory from their own perspective. They believed this was responsible for the large turnout of clients and the cordial provider-client relationship. One respondent said *´quality care includes provision of services such as blood pressure monitoring, weighing, scanning, other laboratories investigations and health education. All these are provided in order to improve the quality of care in the facility´* (49-year-old midwife). Another respondent said, *´we believe that they are satisfied with services rendered to them, because of the massive turnout of clients we receive in this hospital. If they are not satisfied with our services, they will not massively come to this facility*. Another respondent said´ *they are satisfied with the services provided because even in the community people are commenting on our services and sometimes, they show their gratitude to us by thanking us*. (30-year-old CHEW).

**Factors affecting utilization of maternal health services:** common factors reported by the health workers affecting maternal health services in the urban and rural communities include ignorance and lack of knowledge of the importance of antenatal care. Health care providers in the rural communities also complained about late booking and presentation to the facilities mostly when complications develop. One of the discussants said *”Often women cannot decide on where to receive care and need to wait for decisions made by the husband or mother-in-law”*(51 year old midwife). Other factors highlighted include shortage of drugs, equipment or manpower to cater for the large number of clients in both communities. Financial limitations, lack of transportation were further restrictions identified especially in the rural communities.

**Recommendations on improving quality of care:** although majority of the respondents were satisfied with the quality of care in maternal health services in their facilities, recommendations for improving quality of care was an important theme in the discussion. Majority of the discussants felt there is need for improvement in manpower, equipment and supplies. One respondent said *´materials that are distributed to the hospitals for maternal health should also be considered based on population i.e. according to their needs”*.

## Discussion

This study described the quality of maternal healthcare services in the urban and rural communities in Kano State and identified the variation in quality of care received in the communities. The rural respondents had higher number of antenatal care visits of 70.3% compared to 63.5% in the urban communities suggesting in this study that rural respondents had at least 3 ANC visits as recommended by WHO. This is in contrast to the study in Anambra State [13] where more respondents in the urban areas (54.0%) attended ANC up to four times when compared to their rural counterparts (37%). In contrast in a study in Pakistan, only 37% of women reported to have had four or more antenatal visits during their last pregnancy [17]. However, the difference may be due to the larger number of antenatal clients and therefore longer appointments given in the more densely populated urban facilities. Additionally, urban areas are usually characterized by better use of maternal health services given their infrastructural advantages compared to rural areas but similar to another study that confirms this advantage, there was no statistical significance between the two communities as in this study [18]. More patients booked in the second trimester in both urban and rural communities, 59.3% and 64.8% respectively. Similar to this study, a higher median month of booking among rural women was reported in South- Eastern Nigeria [19] and there was also an overall higher prevalence of late ANC booking among rural residents (83.2%) reported in Ethiopia [20]. Late ANC registration and inadequate ANC attendance predisposes the women to higher risks, increased maternal morbidity and mortality and poor pregnancy outcome. Women also lose the opportunity for early detection and effective interventions when complications arise and therefore do not maximally benefit from the services.

Blood pressure measurement, routine investigations and provision of routine drugs were the most offered components of ANC in both communities accounting for 62.6%, 91.3% and 94.9% compared to 92.7%, 94.5% and 96.3% in the urban and rural communities respectively. This was a significant disparity among the communities in relation to the information about warning signs, counseling and tetanus toxoid immunization; 49.3%, 71.2% and 83.1% compared to 57.5%, 49.3% and 55.7% respectively with a statistically significant association for blood pressure measurement, counseling and immunization between the communities. In this study, only 6.8% and 4.1% had used contraception in the urban and rural communities respectively which further supports another study in Nigeria which shows a contraceptive use of 17% and 6% in urban and rural areas respectively [21]. The proportion of women delivered by a skilled birth attendant was higher among the urban respondents (58.4%) compared to their rural counterparts (50.2%). This association was not found to be statistically significant. In a study in rural Kano 96.3% of women had delivered at home or planned to deliver at home [22]. This urban -rural difference is also similar to findings of the National Demographic Health Survey 2018 [22] which revealed births in urban areas are far more likely to benefit from skilled delivery care than those in rural areas. Sixty-six percent of births to urban mothers were assisted by a skilled provider as compared to 29% of births to rural women [22]. This also supports the fact that skilled birth attendance in Nigeria is less than expected when associating ANC attendance with skilled delivery. This is a significant concern in maternal health care delivery as this can influence the outcome of the delivery process, considering the fact that the urban population has easier accessibility to referral services combined with transportation difficulties in the rural areas when the need for referral and immediate intervention arises. However, in a study in Iraokhor [23], Southern Nigeria, a higher proportion of respondents utilized skilled birth attendants at delivery this could be due to the higher level of education in the South compared to the North. This disparity in use of maternal services in various parts of Nigeria, may partly account for the vast difference in maternal mortality in the different regions [24].

Over 80% and more than 70% of the respondents said they had received postnatal care in the urban and rural communities. Conversely, in the study by Jibril *et al*. [25] in Kwara North-Central Nigeria, the authors demonstrated poor attendance in postnatal clinics with means scores of 53.18 for urban and 36.86 for rural communities. The disparity between the findings could be due to the recruitment of respondents from health facilities in this as opposed to the individual households in their study. Respondents in this study are more likely to understand the importance of postnatal care. In another study, the Somali region of Ethiopia had the least postnatal care utilization rate of 3.7% while the capital had the highest utilization at 70%. Women residing in the urban area had 6.4 times higher postnatal care uptake than those living in rural areas [26]. Aside other factors influencing maternal health services, it is believed that the clients´ perception and satisfaction of services determines its utilization. In this study, 70.3% and 65.3% were satisfied with delivery services in their facilities while 9.1% and 14.6% were dissatisfied in the urban and rural communities respectively. Another study in Kano found that were potential patients having access to more than one facility, their perception of the quality of care offered at these facilities often take precedence over concerns about distance [18]. The findings are also similar to a community-based survey in Eastern Nigeria, where Uzochukwu *et al*. [27] found that 94.3% and 95.8% of the women were satisfied with the ANC and delivery services at the health facilities. The respondents in this study received facility care most likely due to their perception of satisfactory services provided leading to a higher proportion of respondents being satisfied with the care received. Qualitative interviews also corroborated this finding as discussants believe the perception of satisfaction with the services is responsible for the large turnout of clients.

## Conclusion

This study found that there is need for improvement in the desirable and minimum acceptable quality of maternal health services in Kano communities. The WHO recommendation of a minimum of at least four ANC visits, early booking, skilled birth attendance, use of contraception and pap smear should be encouraged in both urban and rural communities.

### What is known about this topic

- Quality of maternal services is presumably better in urban areas when compared with rural settings;
- Also, healthcare workers tend to be more interested in staying within the urban areas thereby confounding the shortage of maternal services providers in rural communities.

### What this study adds

- Paucity of data on comparative assessment of rural and urban settings that explore quality of maternal services in northwestern Nigeria;
- It identified that, problems associated with utilization of maternal services are not limited to rural settings in Kano, in addition, availability of trained staff for the provision of maternal services and ignorance are still key challenges in both settings despite various efforts to address that in an attempt to reduce the burden of maternal morbidity and mortality.
